# Experiences of nurses practising mindfulness during self-leadership in delivering a rapid response system for general wards in a private hospital in Gauteng

**DOI:** 10.4102/curationis.v45i1.2298

**Published:** 2022-07-12

**Authors:** Carine J. Prinsloo, Karien Jooste

**Affiliations:** 1 School of Nursing, Faculty of Community and Health Sciences, University of the Western Cape, Cape Town, South Africa; 2 Department of Health Studies, College of Humanities, University of South Africa, Pretoria, South Africa; 3 Department of Nursing Sciences, Faculty of Health and Wellness Sciences, Cape Peninsula University of Technology, Cape Town, South Africa

**Keywords:** critical care, general wards, mindfulness, rapid response system, rapid response team, self-leadership, private hospital

## Abstract

**Background:**

The increased complexity of the nursing care needs of patients and acuity in general wards present nursing care challenges for nurses. Self-led nurses are attentive, taking responsibility for activating the rapid response service when a patient is starting to deteriorate.

**Objectives:**

The purpose of this article is to describe nurses’ experiences practising mindfulness during self-leadership in delivering a rapid response system (RRS) in a private hospital in Gauteng.

**Method:**

A qualitative, exploratory, descriptive and contextual design was followed. Homogenous purposive sampling was used and a total of eight focus groups were conducted. Focus groups durations were between 45 min and 60 min. The data analysis was carried out through open coding on Atlas.ti using the noticing things, collecting things and thinking about things (NCT) approach. An independent coder met with the researcher during a consensus meeting and finalised the analysis.

**Results:**

The findings indicated an underlying theme of nurses being mindful in their self-leadership through the development of self-motivation and self-direction in the RRS. Three categories with six subcategories emerged from the data analysis, namely self-motivation in an RRS by the team, self-direction through role-modelling to peers and training.

**Conclusion:**

Nurses practised mindfulness during self-leadership, utilising behavioural and natural reward approaches and constructive thought patterns. These findings could assist management with developing training programmes for nurses.

## Introduction

A rapid response system (RRS) represents a service where a rapid response team (RRT) brings critical care expertise (Bellomo 2018) to the patient in general wards (low acuity). When a patient exhibits signs of deterioration, such as an elevated modified early warning score (MEWS), the RRT is immediately called to come and assess the patient and provide the necessary treatment with the goal of preventing transferring the patient to a higher acuity level, cardiac arrest or death of the patient (Patient Safety Primer 2019). Globally, the RRS is also known as the critical care outreach service or the medical emergency team. The RRS is a fundamental patient safety system within hospitals. The International Society of Rapid Response Systems state that optimal RRS has the following components: an afferent component to ensure the timely escalation of the deteriorating patient, involving a recognising and alert process for clinical deterioration, and the efferent component for a team-based response to achieve appropriate and timely patient management (International Society for Rapid Response Systems 2021). Nurses spend most of their time providing nursing care activities to patients, including monitoring patients’ vital data; therefore, nurses should be able to detect when a patient starts to deteriorate. According to Purling and King (2012), a nurse’s ability to recognise and respond to signs of patient deterioration promptly plays a critical role in patient outcomes. To attain optimal patient outcomes, nurses need to be aware of and attentive to the patient’s present condition. The global spread of coronavirus disease 2019 (COVID-19) and general ward admissions in hospitals have caused a substantial demand on resources, resulting in a snowball effect, such as unavailable beds in higher acuity level units for a patient who deteriorated in the general ward. As a result, patients with increased acuity remained in general wards, which, in turn, led to increased activation of RRS assistance (Mitchell, Doran, Yuriditsky, Root, Teran, Ma, Shashaty, Moskowitz, Horowityz, Abella et al. 2021).

In South Africa, an RRT is predominantly nurse-led, and the bedside nurse in the ward brings a patient to the attention of the rapid response nurse practitioner, who will assess and treat the patient appropriately or escalate the deteriorating patient to the resuscitation team, which includes a medical practitioner if the deteriorating patient condition determines it. In the RRS, the bedside nurse needs to focus on the patient and the patient’s condition at that moment and not be distracted by thoughts of other patients. The bedside nurse therefore must lead herself or himself by being mindful of the patient’s condition to act appropriately and promptly.

MacKenzie and Baumeister (2015) stated that a mindful self-leader has both high mindfulness and self-leadership abilities, constantly monitors all thoughts and emotions and is aware of all current thoughts, emotions and behaviours. A mindful self-leader acts more consciously and uses self-leadership strategies more effectively than self-leaders who are not mindful (Furtner, Tutzer & Sachse 2018). Self-leadership is upheld when one knows how to be mindful and apply mindfulness strategies (Sampl, Maran & Furtner 2017).

Mindfulness interventions are increasingly used by nurses as an intervention, such as in an RRS, to reduce their workplace stress (Benzo et al. 2018; Braun, Kinser & Rybarczyk 2019). Guillaumie, Boiral and Champagne (2017) defined mindfulness as the practice of remaining focused on present surroundings and activities, for instance when a patient is examined by the RRT, without being distracted by thoughts. Nurses should be attentive to the ‘here and now’ of the needs of deteriorating patients (Kotze 2017) in an RRS. Creswell (2017) defined mindfulness as a process of openly attending, with awareness, to one’s present moment experience. On the other hand, Furtner et al. (2018) defined mindfulness as the planned and non-judgemental self-observation of experiences in the present moment, involving self-regulation of perception and orientation towards experiences in the present moment.

Mindfulness in an RRS has an impact on a stressful situation. According to Arthur et al. (2017), mindful people tend to be more focused and relaxed and can positively manage stress. In recent years, there has been a rise in research activities concerning mindfulness, which indicates the benefits of mindfulness (Carleton, Barling & Trivisnonno 2018; Cheli, De Bartolo & Agostini 2020; Dane & Brummel 2013). Despite the wide implementation of RRS, the literature is silent on RRS nurses’ mindfulness and self-leadership in an RRS.

Self-leadership approaches are needed where nurses in an RRT motivate themselves towards improved performance and effectiveness in nursing patients. Self-leadership amongst nurses is vital for optimal efficiency in nursing practice. Nurses who demonstrate self-leadership qualities will possess those characteristics known to have a positive influence when caring for patients (Holopainen, Nystrom & Kasen 2019).

## Theoretical assumptions

The self-leadership theory grounded this research. The central insight of the self-leadership theory is that the attitudes, beliefs, self-designed behavioural patterns and motivational preferences of individuals (such as nurses) make a critical difference in both accomplishments and personal satisfaction in work (Neck, Manz & Houghton 2020). These self-leadership approaches aim at the improvement of personal effectiveness through (1) behaviour-focused strategies, (2) natural reward strategies and (3) constructive thought patterns (Neck et al. 2020), which are needed in nursing environments.

*Behaviour-focused strategies* can be used to focus the attention of the nurse on their behaviour to promote positive behaviour and exclude undesirable behaviour (Neck & Houghton 2006). Bedside nurses in the RRS focus on their behaviour to call the rapid response nurse promptly when the patient’s MEWS indicates the calling of the RRT. Self-observation, self-goal-setting, self-reward, self-punishment and self-cueing are amongst the behaviour-focused strategies (Neck et al. 2020) that can be used to augment rapid behaviour. Self-observation focuses on the observation of one’s behaviour to gain information about yourself; for instance, whether you know when to call the RRT to assess a patient. Self-observation can lead to nurses directing their thoughts, feelings and behaviours to ensure positive outcomes for patients (Goldsby, Goldsby & Neck 2020), Self-leadership can be seen as a person’s own motivation to achieve the established goals in a certain situation, such as the RRS. Self-motivated nurses are goal-driven to provide the necessary nursing care. Self-reward and self-punishment are used for directed and organised behavioural change (Neck et al. 2020) when a goal is achieved or not achieved. Self-rewarding in an RRS can be when nurses pat themselves on the shoulder when they have focused on the patient’s current condition and performed nursing care activities to prevent the patient from deteriorating.

*Natural reward strategies* should be built into the task itself (Neck et al. 2020). An environment should exist in which the nurse is motivated to perform nursing care activities that recognise their value in the environment. Natural reward strategies are designed to help generate feelings of competence and self-determination, which in turn strengthen performance-enhancing, task-related behaviours (Neck et al. 2020). Manz (2015) stated that self-leaders use this strategy to change their interpretation of their tasks so that the task provides them with feelings of self-control, competence and purpose. Further natural reward strategies include positive interpretations and experiences that can be linked to nurses’ responsibilities, what they believe in, what they are committed to or whether they enjoy the actual work for its own value (Shek et al. 2015). Pramilaa (2016) mentioned that motivation is the energy that inspires a person’s behaviour and performance of tasks.

*Constructive thought pattern strategies* refer to visualising successful performance, engaging in positive self-talk and evaluating beliefs and assumptions to change dysfunctional thoughts (Neck et al. 2020). Engaging in positive self-talk could be seen in reflecting and evaluating one’s own beliefs and assumptions concerning the effectiveness of their performance, that is, the nursing care delivered. The nurse focuses on identifying irrational or dysfunctional beliefs and replacing them with positive thoughts (self-talk) (Goldsby et al. 2020). Sanderson (2017) stated that positive self-talk is self-motivating; therefore, nurses are self-motivated to empower themselves with skills to provide the necessary nursing care. Manz (2015) believed that influencing one’s thoughts to help make one’s thinking more constructive is a critical part of effective self-leadership. Neck, Houghton and Murray (2017) defined self-leadership as a process through which self-leaders regulate their behaviour, influencing and leading themselves through the use of specific sets of behavioural and cognitive strategies.

### Problem statement

The acuity of patients in general wards has escalated considerably over the past years. Patients who were normally cared for in higher levels of care units are increasingly being cared for in general wards (Missen et al. 2018). Delayed response to patient deterioration remains one of the strongest forecasters of mortality and unplanned admission to a higher level of care unit amidst critically ill patients (Ludikhuize et al. 2015). Bedside nurses are seen as the most important team members because they need to identify the patient at risk of deteriorating or who is deteriorating at the moment and then refer the patient to the RRS nurse for personalised interventions (Patient Safety Primer 2019). Patient deterioration is often detected late or missed completely, which contributes to the development of serious adverse events (Peterson, Rasmussen & Rydahl-Hansen 2017). Little research has been carried out regarding what is preventing nurses from activating RRS. Not much is known about nurses’ experience in practising mindfulness in self-leadership in general wards of an RRS at a private hospital.

### Purpose

The purpose of this research was to explore the experiences of nurses in practising mindfulness during self-leadership in delivering an RRS in general wards in a private hospital in Gauteng.

## Research design

A qualitative design was followed to explore and describe nurses’ experiences practising mindfulness during self-leadership in an RRS context.

### Setting

An RRS was initiated for the first time in South Africa in 2005, at a private hospital in Gauteng, which was also the setting for this study. This hospital has nine general wards that used the RRS, including all the nursing level categories working with patients in these wards as part of the RRT.

### Population and sampling

The accessible population in this study was 203 nurses from all three nursing qualification categories, comprising 62 professional nurses, 71 staff nurses and 70 auxiliary nurses who worked day or night shifts in nine general wards. These nurses were RRT members who needed to call the RRS nurse. Homogenous purposive sampling was conducted separately for each of the nursing categories. Focus groups with professional, staff and auxiliary nurses were held separately because of their different scopes of practice, as outlined under the *Nursing Act* (South Africa 2005).

### Data collection

Hospital management permitted the research. Unit managers, acting as gatekeepers, were asked to provide staff with an informational letter explaining the research (benefits, ethical consideration, focus groups), an invitation and a consent letter to participate in the research and digitally record the focus group discussions. Focus groups allowed the researchers to gather rich, detailed information on the phenomenon of self-leadership of nurses in an RRS (Davis 2017).

An interview guide with semistructured questions was used to collect data during focus groups, which were digitally recorded; observational data (field notes) were recorded by a moderator (Creswell & Plano-Clark 2018). The duration of the focus groups was between 45 and 60 min. Eight focus groups were conducted at a convenient time (day and night) in a private room, without any interruptions. Two focus groups with professional nurses and three focus groups with each of the staff nurse and auxiliary nurse groups were held. The participants included 1 male and 56 females, with ages ranging between 22 and 55 years. A total of 11 of the participants were professional nurses (two focus groups), 24 were staff nurses (three focus groups) and 22 participants (three focus groups) were auxiliary nurses. The focus groups had between five and nine participants, after which data saturation was achieved when no new information was collected from participants (Creswell & Plano-Clark 2018). All participants were fluent in English.

### Data analysis

The analysis of the data and field notes were guided by open coding on Atlas.ti using the computer-assisted noticing things, collecting things and thinking about things (NCT) approach (Friese 2019). The NCT data analysis approach does not suggest any specific way of coding but has three basic components, namely *noticing* interesting things in the data, *collecting* these things and *thinking* about them. This method created order, structure, patterns and relations to the volume of data collected. Data were transcribed, read, reread and compared with the field notes to ensure accuracy. Similar interesting experiences were written down, as notes were made and preliminary codes attached. The researcher sought to find patterns in the data and themes and categories developed. An independent coder met with the researcher and reached a consensus on the analysis.

### Trustworthiness

Credibility was obtained through persistent observation and recording of behaviour during the focus groups discussions and answering questions from participants. Dependability was enhanced by digitally recording focus group discussions and dense descriptions of the methodology followed. The discussion guide was piloted to confirm that participants understood the questions and that questions prompted suitable discussions. The steps taken during the data collection and data analysis were documented in a journal and a consensus discussion was held with an independent coder. Participants agreed with the researcher that interpretations and conclusions reflected the voices of the participants, which ensured conformability. The moderator clarified and paraphrased during the focus group discussions, and the discussions were transcribed verbatim. An audit trail confirmed the accuracy of the transcriptions, which further enhanced conformability. Transferability was confirmed when the participants, the research setting, focus group discussions and observations made during focus group discussions were described thoroughly (Creswell & Plano-Clark 2018).

### Ethical considerations

Ethical clearance was obtained from the Senate Research Committee of the University of the Western Cape (reference number 12/7/6), whilst approval was obtained from the private hospital group in South Africa (UNIV-2013-0007B) and the hospital manager to conduct the study. Written informed consent to participate in the study was obtained from participants. The names of the participants did not appear on the transcripts and participants could withdraw at any stage of the research process. No participant had to be referred to a nearby prearranged psychologist, as the focus group discussions entailed minimal risk.

## Results

The findings indicated an underlying theme of nurses being mindful in their self-leadership through the development of self-motivation and self-direction in the RRS, portrayed in the categories and subcategories (Table 1).

**TABLE 1:** Theme, categories and subcategories.

Theme	Categories	Subcategories
Mindfulness in self-leadership through developing self-motivation and self-direction (autonomy) in the rapid response system	1. Self-motivation in an RRS by the team	1.1 Calling the RRS nurse
		1.2 Taking charge and assessing the patient
		1.3 Self-motivated to act as an advocate for patients during doctors’ rounds
	2. Self-direction through role-modelling to peers	2.1 Taking the lead when in charge of RRS nurses
		2.2 Staff involvement in communication
	3. Training	3.1 Mentoring and teaching peers

*Source:* Adapted from Prinsloo, C., 2018, ‘Self-leadership strategies of nurses in an outreach service at a private hospital group in Gauteng’, PhD thesis, School of Nursing, University of the Western Cape, Bellville

### Self-motivation in a rapid response system by the team

Self-motivation occurs when nurses autonomously use strategies to keep themselves on track towards a goal (Pramilaa 2016). Participants express their self-motivation by reflecting on their behaviour, constructive thought patterns and the activation of natural reward strategies.

#### Calling the rapid response system nurse

Self-leadership was demonstrated when they were mindful in applying behavioural strategies, motivating themselves to change their behaviour when they called the RRS nurse to inform them about a patient with an elevated MEWS. An elevated MEWS categorises a patient as being at a high risk of deteriorating, and bedside nurses need to call the RRS nurse to assist them with providing timely quality care to the patient.

It seemed as though nurses debated with themselves (self-observation) about the next step to follow and whether there was a need to call the RRS nurse after they had calculated the MEWS of patients:

‘We’re very observant; you do observations, you monitor them, are they normal or abnormal? Then you can see … what the next step is you can take …’ (FG1, P4, auxiliary nurse)

‘[… *I*]f the patient is complicated, firstly, I can do 123; if there is no progress, I report.’ (FG2, P3, staff nurse)

‘[… *T*]hen also, when you see the patient condition is changing, there is something wrong with the patient, then you just call the outreach [*refering to the RRS nurse*] to come and help …’ (FG2, P6, staff nurse)

Constructive thought patterns were used when nurses had to make a nursing care decision. Neck (2018) stated that there are two general and opposing patterns of thinking, namely opportunity thinking and obstacle thinking. A person who participates in opportunity thinking focuses on constructive ways of dealing with challenges. The nurses are involved in self-talk when they focus their attention on the patient and rethink what they need to do:

‘… I can see that this patient is in danger … it is where I know I can phone the outreach sister [*referring to the RRS nurse*] …’ (FG4, P5, staff nurse)

‘[… *W*]hen you don’t know any more what to do for this patient, you pick up the phone, “Sister [*referring to the RRS nurse*], come help me please …”’ (FG2, P4, staff nurse)

‘[… *Y*]ou know what, as your gut says like this, trust your gut, let’s just do this and this. With us, it helps a lot in paediatric ward ….’ (FG2, P8, staff nurse)

Nurses paid attention to their thoughts when they reflected on the different options of nursing care actions that they could take to deliver appropriate nursing care to the patient.

#### Taking charge and assessing the patient

Constructive thought strategies focused on the nurse’s imagination, motivating herself whilst she paid attention to the patient’s condition and thoughtfully considering what would be the best nursing care task for the patient at that moment:

‘[… *A*]fter observations, you must plan – maybe that blood pressure is high. Like she said, then [*the*] patient can be in pain, but not verbalising he is in pain, then if you can give something for pain, then the blood pressure can go down, and then after, yes, it can go down …’ (FG1, P3, auxiliary nurse)

‘Sometimes at some stages, you find that the patient is presenting with some signs and symptoms but the others are normal, others are abnormal, that is when you find that you are in-between; you are confused, you are not sure if this could be that, or it could be, so you start thinking but something is wrong with this patient, because they are not correlating together, like this one is normal but there is something else that is abnormal when you check the patient.’ (FG7, P9, staff nurse)

‘You call … ja when you feel like you can’t pinpoint [*P8*: *nodding*] that something wrong is wrong or happing to the patient, you call someone else and you include them, [*to come and check*] to help you evaluate when you together and then stuck, [*indicating that they don’t know how to manage the patient*] and then calling the outreach sister [*referring to the RRT nurse*]. You see something is going on, but you can’t pinpoint what is going on, then maybe the sister, the outreach sister [*referring to the RRT nurse*] with more knowledge, with more experience will be able to help you to get the things together.’ (FG7, P1, staff nurse)

Ein-Gar and Steinhart (2017) stated that self-control refers to intentional regulated actions in an attempt to achieve goals. Measuring a patient’s vital observations could become a routine task and nurses need to exhibit self-control and be mindful when measuring a patient’s vital data. Nurses motivated themselves when they mindfully thought ahead about which actions they needed to take around required nursing care. Effectively taking charge of deteriorating patients created a feeling of competence and self-control amongst nurses which, in turn, was naturally rewarding to the nurse. Participants mentioned how they considered (mindfully) what would be the best action for a deteriorating patient:

‘You must make decisions, because if you are there [*four others nodding*], you are alone then – okay, my patient, the blood pressure is very high, what is my next step? For example, I am using a dinamap, the blood pressure is 200 over what, what, what. Now, okay, my next step is I am going to take the manual one … and check it again. I took one step, second step is this, then now, okay, there is something wrong with this patient.’ (FG1, P2, auxiliary nurs)

‘[…*W*]hen you do his observations you see everything if it is going down and action can be taken … and it is there where you see now I am doing the right thing …’ (FG4, P5, staff nurse)

‘For instance, if the patient is from theatre and then you see that patient, the blood pressure is going down, the patient is pale, then you know the first thing you must do is Hb. At least when the outreach sister [*referring to the RRS nurse*] comes, she is having a baseline on what to do …’ (FG1, P3, auxiliary nurse)

When checking a patient’s vital data, the nurse becomes aware if the data are abnormal. The participants explained how they focus on taking charge of the patient when dealing with abnormal vital data and being motivated to implement actions to respond to such data, which, in turn, increases the sense of self-determination and autonomy (a natural reward). Self-determination involves the belief that one has control, choice or autonomy over one’s work behaviours and processes (Ryan & Deci 2017).

#### Self-motivated to act as an advocate for patients during doctors’ rounds

Participants (staff nurses) accompanied doctors during rounds as they were empowered to do so. It enhanced their self-perception and increased their sense of competence:

‘[… *W*]e said to them that they must do rounds with doctors, and the doctors must accept it, that’s how it is. We cannot – we work with one sister on a shift, then we have 32 patients and it doesn’t work, so the doctors know now, it is discuss[ed] with them, and all of them together …’ (FG2, P2, staff nurse)

‘[… *W*]hat we do in our ward, if the sister is doing rounds with the doctor, the EN [*staff nurse*] and ENA [*auxiliary nurse*] working with those patients must escort you, so if the doctor wants to ask something they are there, then he can ask them, and they can answer …’ (FG2, P1, staff nurse)

Escorting doctors on their visits to patients, being mindful of the doctors’ procedures and asking questions related to the patients’ illness and treatment could create a learning experience for nurses, which increases their feeling of competence (a natural reward) that could motivate nurses to advocate for patients. Ross (2014) and Manz (1986) believed that when individuals overcome challenges, the self-perception of personal competence and efficacy increases and the individual becomes more self-confident.

### Self-direction through role-modelling to peers

Morgenroth, Ryan and Peters (2015) suggested that role models motivate individuals to set and achieve goals. Garcia-Martinez (2021) stated that it is crucial to be mindful of your behaviour and actions because it can motivate and empower others and help them to grow professionally. Nurses should be mindful that they are role models to other nurses and should be aware of their behaviour and attitude when they take control of a deteriorating patient.

The nurse takes control confidently when the patient is deteriorating and is motivated to visualise what tasks are needed to be performed when taking care of a patient. Nurses create a mental image (using mental imagery) of the desired outcomes or goals and make a thoughtful decision to nurse the patient without delay to achieve the desired objective (Neck & Houghton 2006; Neck et al. 2017). Whilst being mindful, one is aware of the moment without holding onto automatic reactions (Bernstein 2019). Nurses are mindful when they take control and thoughtfully make decisions about the nursing care of a deteriorating patient.

#### Taking the lead when in charge of calling the rapid response system nurse

Participants regarded themselves as role models (granting themselves self-efficacy). As role models, they were motivated to involve their peers (the bedside nurses) in nursing a deteriorating patient, which empowered the bedside nurses:

‘My authority, I think, is to make sure that the patient’s condition is handled correctly, not to deteriorate but for improvement. So if she needs to be transferred, she must be transferred urgently. So I have to be firm that everything is carried out quickly, not delayed or being negligent …’ (FG1, P3, auxiliary nurse)

‘[… *B*]eing the leader taking control, going … maybe to the shift leader … getting her input as well and reporting back to the staff, because doesn’t help they come and report and you have to go back to him …’ (FG4, P7, staff nurse)

‘[… *Y*]ou know what, I just feel like … we can do something … we don’t have to wait for the seniors, you know – “I can’t do anything because I am ENA …”’ (FG8, P7, auxiliary nurse)

Another participant practised self-goalsetting by striving to be the best and being a role model to other nurses:

‘… I should expect more of myself and I should show them what I expect of me before I can expect anything from them, so that is what it is all about to me, because this role model that I have had … the juniors looking up to me as I am looking up to her. So for me, it is a personal thing about being the best that I can be in the situation that I am and it is about respect.’ (FG3, P2, professional nurse)

Lyons and Bandura (2018) maintained that self-efficacy is to a great extent positively connected to individual motivation, self-confidence, proactive behaviour and work performance. It could be assumed that nurses’ self-confidence in their competence to take control of a deteriorating patient will motivate them to provide the appropriate nursing care for such a patient. Bandura (1991) explained that self-efficacy through self-regulation is a mindful self-management system and comprises a process of guiding your actions and thoughts to achieve one’s goals. In order to uphold self-efficacy, bedside nurses should thoughtfully (mindfully) guide their behaviour in achieving the goal of delivering suitable nursing care interventions to prevent the patient from deteriorating.

#### Staff involvement in communication

Communication between healthcare workers is a daily activity and is a vital part of providing appropriate patient care. Clear and effective communication between healthcare workers can be seen as a facet of self-leadership (Holroyd 2015). Furthermore, to ensure teamwork between healthcare workers, quality communication is required (Lymberakaki, Sarafis & Malliarou 2021).

‘[… *I*]t is all about communication, about communicating to the staff and telling them what your expectations are. When you do orientation, make sure that every new member in the ward gets orientated, and a proper orientation. Then it is also about repetition, not saying it once to them and think that they are going remember all of that …’ (FG3, P2, professional nurse)

The participants indicated that nurses need to utilise effective communication skills to ensure that all healthcare workers know what is expected of them. They need to assist one another in providing the necessary nursing care tasks correctly when their patients need them:

‘[… *Y*]ou can also involve the staff with whom you are working with. As an EN [*staff nurse*], I can also talk to my ENA [*auxiliary nurse*] so that they can report each and every patient, every intervention that is because of the patient …’ (FG4, P1, staff nurse)

‘[… *B*]ut you need to acknowledge first that you do not know everything, the same way that I do, and then you ask if you [*are*] not sure, you must communicate. You communicate with the patient, you communicate with other staff members, we communicate with the doctors, we communicate with everyone …’ (FG3, P1, professional nurse)

### Training

#### Mentoring and teaching peers

According to Van Dam (ed. 2018), providing employees with opportunities to learn and develop new competencies involves healthcare workers acquiring skills:

‘[… *W*]e do a lot of training. I mean we do, there is a sister who does bloodgas training so you know for this little ones – I mean, when these young professional nurses finish, it is good to go for a course on … a little bit more advanced, like reading of ECGs [*electrocardiograms*], understanding bloodgasses and all those things and we do …’ (FG2, P4, staff nurse)

‘[… *A*]nd also to encourage more training at it (hmmm), updates, how things are done, even if it is not a permanent staff …’ (FG4, P4, staff nurse)

Training provides participants with an increased sense of competence, which supports their self-leadership as natural rewarding, which is in line with the statement by Neck et al. (2017) that work is more naturally rewarding (self-leadership strategy) when a task delivers a sense of competence.

## Discussion

Practising mindfulness in self-leadership, nurses seemed to use self-motivation and self-direction in an RRS in a private hospital in Gauteng. Overall, nurses, as part of the RRT, experienced the successful interventions that they provided to a deteriorating patient as self-motivating.

There is clear evidence that self-motivated nurses focused their attention on their own behaviour to adopt positive behaviour, providing nursing care interventions for a deteriorating patient (Neck et al. 2020). Nurses purposefully paid attention (mindfully) to how they should behave to manage a patient with an elevated MEWS. The bedside nurses were motivated to attain the goal of providing nursing care interventions to a patient who was deteriorating when they used the MEWS as a cue to call the outreach expert nurse to assist them with the nursing management of a patient with an elevated MEWS (Neck et al. 2020). The bedside nurse in the general ward set herself or himself the goal to call the RRT for assistance with a deteriorating patient (Bellomo 2018). Self-observation was implemented to determine the conditions for using certain behaviours such as when a nurse calls the RRT when she lacks the skills to manage a deteriorating patient. Neck et al. (2020) believed that self-observation forms the foundation for certain behaviours. Nurses demonstrated their mindfulness when they focused on behaviour that was needed to manage a patient with an elevated MEWS. Iqbal and Nadeem (2018) indicated that a positive relationship exists between mindfulness and job performance.

Participants needed to take charge of and assess the patient. The participants indicated how they had to think ahead (using mental imagery) about the different actions to take for patients. They were mindful when they focused on adapting their behaviour to attend to the care of a deteriorating patient. Mindfulness is defined as being able to provide uninterrupted attention to any task, without judgement or criticism (Siegel 2016).

Constructive thought strategies, as outlined by Manz (1986), were observed as participants increased their effectiveness by facilitating the management of thought patterns. Practising mindfulness delivers a time-based period to pause and reflect upon more thoughtful decisions (Huang 2017). The nurse who focuses on visualising successful performance is seen as visualising what interventions are needed to achieve the desired outcome (Neck et al. 2020), for instance, initiating the RRT when the patient is starting to deteriorate.

Nurses are mindful when they are aware of the positive feelings of competence, self-determination and autonomy they experience whilst providing nursing care to a patient with an elevated MEWS. Bernstein (2019) mentioned that by being mindful, one is aware of one’s views, physical sensations and feelings without getting caught up in instinctive responses or positive or negative judgements. These positive feelings are naturally rewarding to a nurse who takes charge (self-determination) of a patient with abnormal vital data (elevated MEWS), which generates feelings of competence (Manz 1986, 2015). Evidence showed that the nurses felt it enhanced their self-perception and increased their sense of competence, a natural reward (Neck et al. 2020), when they accompanied doctors on their rounds visiting their patients. One of the four facets of mindfulness as described by Baer, Smith and Allen (2004) is to ‘act with awareness’ and refers to paying focused attention to one’s efforts. The nurses indicated how they, as role models, were motivated to empower their peers when they involved them in nursing a deteriorating patient. The nurses as role models focused on sharing their knowledge with their peers, and this created feelings of competence (Neck et al. 2020) amongst the nurses.

The nurses felt that they needed to engage in effective communication to ensure that they knew what was expected of them. Holroyd (2015) stated that communication is a facet of self-leadership and higher levels of perceived self-efficacy go together with higher performance accomplishments where communication is considered to be a component (Schwarzer 2014). Focusing on these activities may form part of better-quality training.

There is clear evidence that training provides employees with the competencies they need in delivering nursing care to their patients, which builds competence and motivation (Tarique 2014).

The findings showed that the nurses initiated constructive thought patterns when they visualised which tasks needed to be performed when taking care of a patient with an elevated MEWS (Dizaho, Salleh & Abdullah 2017). These findings suggested that participants were mindful when they were aware of the feelings of competence they experienced during nursing care activities. Nurses also made thoughtful decisions to achieve the desired outcomes for the patient and did not respond instinctively to a patient’s condition. Rather, they consciously paid attention to the patient’s vital data and thoughtfully decided what the best action was.

## Limitations of the study

Rapid response systems are not generally implemented in South Africa, and this study was conducted using participants from one private hospital; thus, the findings from this study cannot be generalised to other hospitals.

## Recommendations

Nurses should focus on being mindful of their self-leadership through developing self-motivation and self-direction in an RRS to improve the health outcomes of patients when they are providing nursing care activities to deteriorating patients. Nurse managers should encourage ward nurses to be mindful when providing nursing care to patients and not be distracted by other thoughts or activities.

It is recommended that organisations should focus on empowering nurses. Empowering nurses through training will increase nurses’ competence in providing nursing care for patients. Neck et al. (2017) stated that the feeling of competence is naturally rewarding. Refreshing and maintaining nurses’ skills are important to keep them up to date with innovations and healthcare developments and will provide nurses with the expertise to call outreach nurse experts for assistance with providing nursing care to a deteriorating patient. Training nurses towards self-leadership should be increased. This could be carried out through self-leadership workshops, which would provide nurses with the necessary skills to effectively apply self-leadership strategies when providing nursing care to patients. Nurses will have the ability to develop themselves through self-motivation and self-direction, which will lead to higher performance and effectiveness (Neck et al. 2020). Training of nurses should focus on competencies that nurses need for caring for a patient who is at risk of deteriorating or started deteriorating, for instance, calculating MEWS. Nurses should be knowledgeable and understand how to calculate MEWS and, during training, the importance of vital data monitoring should be emphasised, which will contribute to nurses’ self-leadership.

Organisations should create a mentor–mentee programme to empower nurses. Nurses as role models (mentors) could empower their peers. A mindful mentor would provide advice to a mentee using their existing knowledge, skills and experience in order to advance their professional performance and development (Garvey, Stokes & Megginson 2018). Lastly, the researcher recommends that an RRS should be implemented in private and public hospitals.

## Conclusion

Mindfulness in self-leadership was applied through behavioural and natural reward approaches and constructive thought patterns. Nurses being mindful of their self-leadership in the outreach service could create self-motivated nurses who are proactive in the nursing care and management of patients with an elevated MEWS or who are deteriorating. Nurses’ experiences practising mindfulness during self-leadership in an RRS may assist management with developing training programmes for nurses. Training of nurses should focus on improving nurses’ competency in providing nursing care to deteriorating patients which will, in turn, contribute to nurses’ self-leadership strategies.
